# Neuroprotective effert of carbon monoxide and Nrf2 in cerebral ischemia

**DOI:** 10.1186/2193-1801-4-S1-L44

**Published:** 2015-06-12

**Authors:** Sylvain Doré

**Affiliations:** Center for Translational Research in Neurodegenerative Disease, University of Florida, College of Medicine, USA

**Keywords:** Medical gas, Neuroprotection, Nrf2-regulated protein, Stroke

Carbon monoxide (CO) is a gaseous second messenger produced when heme oxygenase (HO) enzymes catabolize heme. We have demonstrated that CO can be therapeutic in ischemia-reperfusion brain injury; however, it is unclear whether CO can also offer protection in permanent ischemic stroke or what mechanism underlies the effect. HO1 neuroprotection is shown to be regulated by Nrf2; therefore, we investigated whether CO might partially exert neuroprotection by modulating the Nrf2 pathway. To evaluate potential protective effects of CO, we exposed wildtype and Nrf2-/- mice to 250ppm CO or control air for 18h immediately after permanent middle cerebral artery occlusion. Infarct volume and neurological deficits were assessed on day 7. Molecular mechanisms of Nrf2 pathway activation by CO were also investigated. Mice exposed to CO after permanent ischemia had 29.6±12.6% less brain damage than did controls at 7d. Additionally, 18h CO treatment led to Nrf2 dissociation from Keap1, nuclear translocation, increased binding activity of Nrf2 to HO1 antioxidant response elements, and elevated HO1 expression 6-48h after CO exposure. The CO neuroprotection was essentially completely abolished in Nrf2-/- mice. Low-concentration of exogenous CO represents a neuroprotective agent for stroke combination treatment and its beneficial effect would be at least partially mediated by activation of the endogenous Nrf2 pathway.

